# Effects of sweet chestnut (*Castanea sativa Mill*.) hydrolysable tannins on animal performance, health and manure ammonia and nitrous oxide emissions in preweaning holstein calves

**DOI:** 10.3389/fvets.2026.1868627

**Published:** 2026-08-14

**Authors:** Elena Scaglia, Laura Gogna, Anna Antonioli, Livio Galosi, Gianni Gilioli, Valentina Caprarulo

**Affiliations:** 1 Agrofood Research Hub, Department of Civil, Environmental, and Architectural Engineering and Mathematics (DICATAM), University of Brescia, Brescia, Italy; 2 School of Biosciences and Veterinary Medicine, University of Camerino, Matelica, Italy; 3 Istituto Zooprofilattico Sperimentale della Lombardia e dell’Emilia Romagna “Bruno Ubertini”, Brescia, Italy

**Keywords:** ammonia reduction, calves diarrhea, dairy farm, environmental sustainability, feed additives, greenhouse gas, gut health

## Abstract

Tannins are plant-derived compounds that can improve animal health by modulating gut health, and reducing the incidence of diarrhea, also influencing the emissions of nitrous oxide and ammonia in animal manure. This study evaluated the effects of hydrolysable tannins from sweet chestnut (*Castanea sativa Mill.*) on growth performance, intestinal health, clinical insights, and ammonia and nitrous oxide emissions from manure in Holstein calves. Twenty-four preweaning female Holstein calves were randomly assigned to two experimental groups according to body weight (BW; 39.37 ± 3.42 kg) and age (3.83 days-of-life) for a 56-day trial, a control group (CTRL) fed with milk replacer, and a treatment group (VITAN) fed milk replacer supplemented with 10 g/day of chestnut tannins. Body weight and rectal temperature were recorded on d 0, 7, 14, 21, 28, 35, 42, 49, 56. Milk intake, hay intake, average daily feed intake of solid feed, average daily gain and fecal and clinical scores were daily recorded. Fecal samples were collected on d0, d28, and d56 to determine total bacterial counts, coliforms and *Lactobacillus* spp. Blood samples were collected on d0 and d56 for metabolic profile. After the *in vivo* trial, manure (feces, urine and straw) of CTRL and VITAN were collected from individual pens, and pooled into six dynamic-chambers (3/treatment) to simulate a 49-day manure storage period. Emissions of NH_3_ and N₂O (mg/g/h) were monitored by Gasmet-GT5000, and chemicals analysis were performed on d0 and d49. VITAN group showed a reduction of solid average daily feed intake (*p* < 0.01) without compromising growth performance. The VITAN group showed significantly higher frequency of normal fecal consistency (FS0-1; *p* < 0.01) and improved fecal consistency. Tannin supplementation appeared to influence manure emissions, lower NH₃ emissions were observed on d0 and 1, and lower N₂O emissions on d1, 2, and 5 in VITAN group. Manure temperature was higher in VITAN group. Chemical analysis also showed lower total solids, ash, and total carbon in VITAN manure on d49. These results should be considered preliminary and require confirmation in studies including biological replicates and appropriate statistical evaluation. In conclusion, tannins might represent a promising nutritional strategy with potential effects on health-related parameters and manure gaseous emissions under the conditions evaluated in this study.

## 1 Introduction

In dairy herds, animal health is essential to ensure welfare and maximum productivity and economic return. Among the different stages of life, the pre-weaning period represents one of the most critical phases in a calf’s life due to the high susceptibility to diseases, the ongoing physiological and metabolic development, and its long-term impact on growth performance and future productivity (1). Among young calves, diarrhea remains a leading cause of morbidity and mortality, often prompting the use of antibiotics (2). To address this critical issue, feed additives may be considered as a complementary strategy to reduce disease incidence and limit antimicrobial use (3). Feed additives can positively influence animal health while also enhancing nutrient digestibility and feed efficiency. Moreover, they may modulate the gut microbiota, reduce nitrogen and phosphorus excretion, mitigate gaseous emissions, and improve growth performance. Overall, their use represents a promising strategy to increase production efficiency while promoting environmental sustainability and reducing reliance on antimicrobial treatments (4). Improvements in nutrient utilization efficiency may decrease the excretion of nitrogen and carbon, thereby potentially reducing the substrate available for microbial processes responsible for gaseous emissions during manure storage (5). From an emissions perspective, the European Union has set ambitious targets to be achieved by 2030, placing environmental sustainability among the key priorities, particularly within the livestock sector (6). Livestock production systems contribute significantly to agricultural nitrogen emissions, including nitrous oxide (N₂O) and ammonia (NH₃), making them a key component in the assessment of the nitrogen cycle. In this context, considerable research has focused on plant secondary metabolites such as tannins, which are known to bind dietary proteins and thereby alter nitrogen metabolism, utilization efficiency, and excretion patterns (7, 8). Nitrogen excreted in urine and feces represents a major source of reactive nitrogen emissions, leading to the formation of NH₃ and N₂O.

Feed ingredients and additives have multiple positive functions, including the enhancement of environmental sustainability of animal production. Effective mitigation strategies are crucial to maintaining herds sustainability, and additives play a key role in these strategies (9). Tannins are polyphenolic secondary plant compounds capable of forming complexes with proteins and carbohydrates through hydrogen bonding. Owing to these chemical proprieties, they have gained attention for their potential benefits in ruminant nutrition for improving health, indirectly influencing gas production, and serving as bioactive compounds with multiple beneficial effects (9). Tannins can be divided in two major groups, hydrolyzed and condensed tannins (10, 11). Particularly, hydrolysable tannins are characterized by esterified phenolic groups, originating gallotannins and ellagitannins and their structure may vary under specific processing conditions, such as thermal processing, esterification, and acid or base treatment (10–14). This additive have been studied in many animal species for their multifactorial properties, including antioxidant, antibacterial, antiviral, antiparasitic, antidiarrhea, and anti-inflammatory activity and the biological effects of tannins can be explained in two principal properties, the binding properties in the gastrointestinal tract, and absorbable tannins that show a systemic effect (13, 14). The binding action of tannins causes the precipitation of specific salivary proteins, particularly proline-rich proteins (PRPs) (11). This interaction, driven by hydrophobic forces and hydrogen bonds, is a key defense mechanism that creates the sensation of astringency and is associated with several beneficial effects on animal health (15). In calves, tannins have been studied for their ability to reduce diarrhea (16), enhance growth performance and metabolic antioxidant capacity, reducing the quantity of pathogens in feces, resulting in healthier animals (17), a condition that remains a major cause of morbidity and economic losses in pre-weaned calves (18). Beyond their nutritional benefits, tannins play a significant role in mitigating the environmental nitrogen impact of livestock production (10). Specifically, tannins have consistently demonstrated a reduction in NH_3_ emission from slurry manure (19). The effects of tannins on calf health and performance have been investigated in several studies (16, 17, 20). However, their potential role in reducing manure emissions and nitrogen losses in preweaning calves warrants further investigation. We hypothesized that supplementation of milk replacer with hydrolysable tannins from Sweet Chestnut (*Castanea sativa Mill.*) would improve fecal consistency and health status, modifying manure chemistry, and decreasing ammonia and nitrous oxide emissions in preweaning Holstein calves. By integrating animal health and environmental sustainability metrics of nitrogen, this research seeks to provide an integrated approach to sustainable calf rearing, offering potential contribution to prevention strategies while mitigating livestock’s ecological impact.

## 2 Materials and methods

### 2.1 Animals, treatments, and experimental design

The study was conducted in a commercial dairy farm located in the province of Mantova (Italy). The trial was approved by the Animal Welfare Body of the University of Camerino (number 6/2024). The study population comprised 24 healthy preweaning female Holstein calves with standardized initial body weight (BW; 39.37 ± 3.42 kg) and age (3.83 days of life), the *in vivo* trial lasted 56 days. Day 0 was considered the first day of tannin supplementation, and calves remained in the trial for the following 56 days. All personnel involved in animal handling and daily scoring were trained prior to the study. The inclusion criteria for animals considered were based on age (2–7 days of life), colostrum quality (>25 brix), and colostrum quantity intake (4 L, from own dam or the colostrum bank, within 6 h after birth) administered via esophageal feeders. Calves were blocked according to BW and age, and then randomly allocated into two groups, a control group (CTRL; *n* = 12) received milk replacer (MR; Milka Evo 30, Cima breeding srl) (Table 1), and a treatment group (VITAN; *n* = 12), fed MR with 10 g/day of chestnut tannins, corresponding approximately to 0.20 g/kg BW/day. The supplementation level was selected based on the protocol described by Bonelli et al. (16), who administered 10 g/day of chestnut tannin extract to pre-weaned calves throughout the experimental period. Calves were housed in individual boxes with straw beds under identical management conditions. Upon trial initiation, a standardized feeding plan was adopted, calves received MR twice daily, starting at 4 L/d and increasing by 1 L per week to a maximum of 8 L/d. The MR powder, 140 g/L was dissolved in water and served in a bucket at 39 °C. Ten grams of chestnut tannin was homogenized into the milk of the VITAN group using a whisk and administered during the morning feeding and maintained throughout the entire trial period (from d 0 to d 56). Tannins (SaviotaN® Feed Powder) derived from chestnut wood containing 224.8 mg/g of total hydrolysable tannins, with vescalagin (45.2 mg/g) and castalagin (39.7 mg/g) representing predominant compounds. Ellagic acid and gallic acid concentrations were 7.8 mg/g and 17.0 mg/g, respectively (batch number 463521). The chemical composition of the tannin product used in the trial was characterized by 6.9% moisture, 1.9% crude protein, 1.5% ash, 0.7% starch, and 0.9% crude fiber. Solid feed (SF; Top Starter TXT, Purina) (Table 1), straw, and water were offered *ad libitum* from day 0, all served in individual buckets. The weaning process began after the end of the experimental period, when calves were transferred to group pens and milk replacer allowance was gradually reduced according to standard farm management practices. Calves were housed in individual pens (1.2 m × 2.0 m; 2.4 m^2^ per calf) arranged side-by-side with perforated metal bar walls to allow direct visual and tactile contact. The space allowance exceeded the minimum requirement of 1.5 m^2^ per calf (Directive 2008/119/EC). Extended individual housing beyond 8 weeks of age was authorized by the farm veterinarian, as permitted under Article 3(1)(a) of Directive 2008/119/EC, and approved by the Animal Welfare Body (OPBA) of the University of Camerino (protocol number 6/2024). All animals were under daily veterinary supervision. After the 56-day experimental period, calves were transferred to group pens (4 m × 4 m; 4–6 calves per pen). The sample size was approved by the Animal Welfare Body (OPBA) of the University of Camerino (protocol number 6/2024) in accordance with Directive 2010/63/EU and the 3Rs principle. For fecal bacterial counts, samples were collected from 6 calves per group (12 calves total), as approved by the OPBA to minimize animal handling and stress. Blood sampling was integrated with routine farm management practices, as residual blood from serological analyses performed by IZSLER was recovered for metabolic profiling, thus not representing an additional invasive procedure.

**Table 1:** Nutritional composition of milk replacer (MR) and solid feed (SF) (data provided by producer: CIMA breeder Srl and Purina).

Item	MR^1^ (% DM basis)	SF^2^ (% DM basis)
Crude protein	22	19
Ether extract	17	3.5
Crude fiber	—	5.15
Ash	8.5	6.85
Lys	2	—
Met+Cys	0.8	—
Ca	0.9	—
P	0.8	—
Na	0.7	0.21

1 Additives per kg, nutritional additives: vitamin A 25,000 UI, vitamin D3 5,000 UI, vitamin E 200 mg, vitamin K3 3 mg, vitamin C 200 mg, vitamin B1 9 mg, vitamin B2 8 mg, vitamin B6 7 mg, vitamin B12 0.05 mg, niacin 70 mg, acid D-panthotenic 26 mg, colin 300 mg, Fe(II) solfate-monohydrate 100 mg, potassium iodate 1 mg, Cu-sulfate pentahydrate 5 mg, Mn-sulfate monohydrate 50 mg, Zn-sulfate monohydrate 50 mg, sodium selenite 0.25 mg, technological integration: butylhidroxitoluene 35 mg.

2 Additives per kg, vitamins, provitamins, and substances with similar effects: vitamin A 23,205 UI, vitamin D3 3,250 UI, vitamin E 79 mg, vitamin B1 26.7 mg, vitamin B2 13.3 mg, vitamin B6 13.3 mg, vitamin B12 0.31 mg, niacin 107 mg, calcium D-panthotenate 26.7 mg, biotin 0.054 mg, choline chloride 180 mg, Beta carotene 1.3 mg. Trace element compounds: copper sulfate pentahydrate - Cu 28.4 mg, iron(II) sulfate monohydrate - Fe 51.2 mg, anhydrous calcium iodate - I 2.3 mg, manganese oxide - Mn 137 mg, sodium selenite - Se 0.57 mg, zinc oxide - 171 mg.

### 2.2 Zootechnical evaluation, fecal and blood sampling

**Figure 1:**
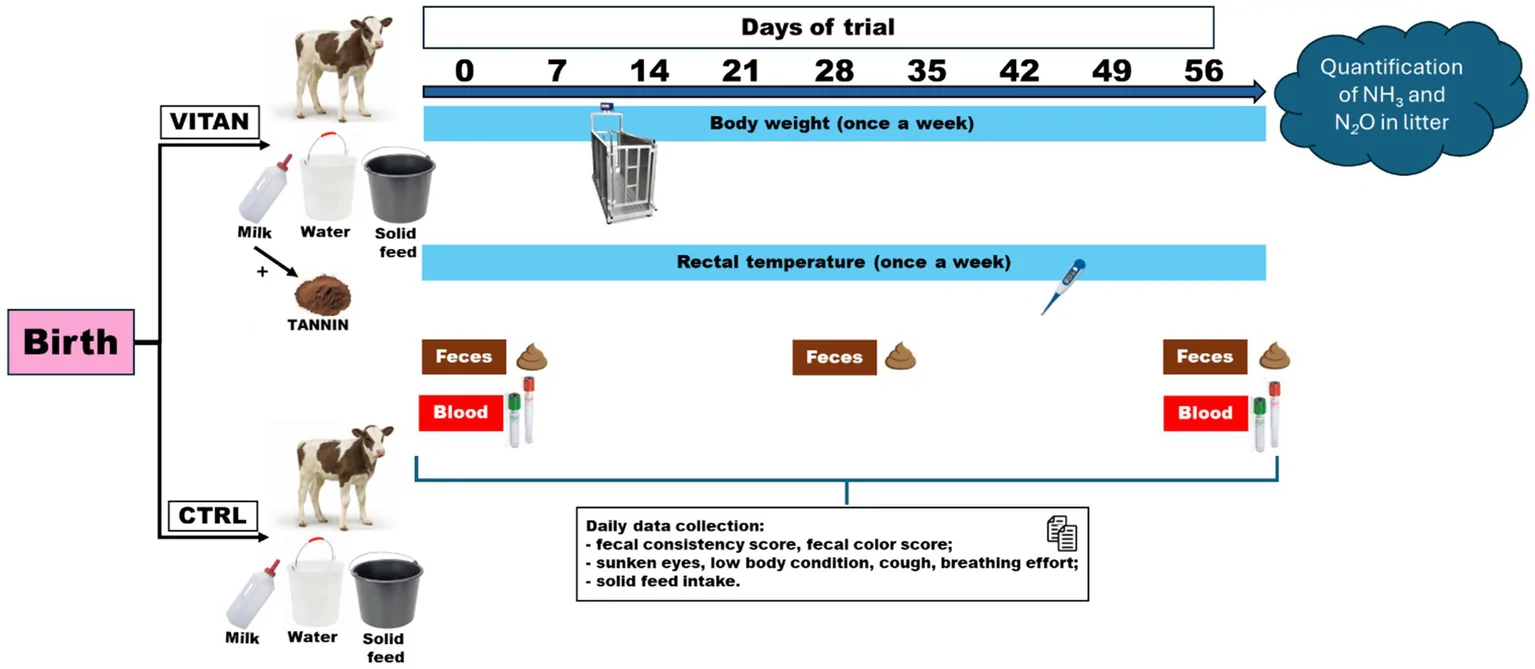
Experimental design of the trial.

Calves were weighed individually, and their rectal temperature (RT) was recorded on d 0, 7, 14, 21, 28, 35, 42, 49, 56 (CTRL and VITAN group). BW of the animals was monitored weekly, and the average daily gain (ADG) was calculated on d 7, 14, 21, 28, 35, 42, 49, 56. Milk intake (MI), hay intake (HI) and average daily feed intake of starter intake were recorded daily (ADFI, g/as fed) was calculated. Fecal samples were collected on d 0, d 28 and d 56. ‘For routine health monitoring, one blood sample was collected individually via the jugular vein on d 0 and d 56; a residual aliquot was used for the purpose of this study (Figure 1), before the morning milk meal, using both lithium-heparinized Vacutainer tubes for plasma analyses and plain Vacutainer tubes without anticoagulant for serum analyses. (BD Vacutainer; BD and Co., Franklin Lakes, NJ). Heparinized tubes were immediately placed on ice and centrifuged at 3,000 rpm for 15 min at 4 °C upon arrival at the laboratory to obtain plasma. Tubes without anticoagulants were allowed to clot at room temperature before centrifugation under the same conditions to obtain serum for protein electrophoresis.

### 2.3 Fecal and clinical score evaluation

Fecal consistency score, fecal color score (21), and clinical sign score (22) were daily monitored for 56 days by trained personnel. Before the beginning of the trial, all personnel involved in the scoring procedures received specific training to standardize the assessment criteria and reduce inter-observer variability. The tannin additive was administered directly by the farm staff, whereas scoring activities were performed exclusively by the trained research personnel. Fecal consistency score was included in the trial to evaluate the daily fecal consistency of the animals. The score ranged from 0–3, where 0–1 scores represented normal fecal consistency (0 = firm but not hard, 1 = not hold form, piles but spreads slightly), while 2–3 scores indicated a low fecal consistency (2 = spreads readily, mild soft diarrhea, 3 = liquid consistency, splatters, severe diarrhea). Fecal color was scored as 0, 1, and 2, corresponding to brown, yellow, and green, respectively (21). The clinical sign scoring system incorporated multiple parameters, including sunken eyes (0–4 score), low body condition (0–5 score), cough (0–2 score), breathing (0–1 score) and diurnal temperature fluctuation. Sunken eyes were assessed through visual inspection and palpation of the temples to determine dehydration severity (0 = normal condition, 1 = slightly sunken temples, 2 = slightly sunken temples and eyes, 3 = moderately sunken temples and eyes, 4 = severely sunken temples and eyes). Low body condition score was evaluate based on animal growth, muscle development, coat quality, and signs of lethargy or rickets (0 = normal condition, 1 = small calf, 2 = small calf with poor muscle development, 3 = mall calf with poor muscle development and rough coat, 4 = small calf with poor muscle development, rough coat, and stunted growth, 5 = small calf with poor muscle development, rough coat, stunted growth, and weakness). Cough (0 = no cough, 1 = intermittent cough, 2 = continuous cough) and breathing (0 = normal breathing, 1 = difficult breathing) were evaluated through visual observation of the animals (22).

### 2.4 Plasma metabolic profile

To evaluate the metabolic effects of tannins, plasma and serum parameters were analyzed. Plasma samples were analyzed to determine albumin (g/L), globulin (glob; g/L), albumin/globulin ratio (alb/glob ratio), urea (mmol/L), non-esterified fatty acids (NEFA; mmol/L), beta-hydroxybutyrate (BHB; mmol/L), glucose (mmol/L), total cholesterol (mmol/L), high-density lipoprotein (HDL; mmol/L), triglycerides (mmol/L), aspartate aminotransferase (AST-GOT; mmol/L), gamma-glutamyl transferase (GGT; IU/L), total bilirubin (μmol/L), creatine kinase (CK; IU/L), creatinine (μmol/L), alkaline phosphatase (ALP), Ca (mmol/L), P (mmol/L), Mg (mmol/L), using enzymatic colorimetric methods validated under ISO/IEC 17025 accreditation (23), performed with a multiparametric clinical chemistry autoanalyzer (ILab 650; Instrumentation Laboratory Company, Lexington, MA, United States). Serum protein electrophoresis was performed to determine total protein (g/L), *γ*-globulins (g/L), using an agarose gel electrophoresis system (Hydrasys, Sebia, Lisses, France). All analysis were performed by the Istituto Zooprofilattico Sperimentale della Lombardia e dell’Emilia-Romagna “B. Ubertini,” Brescia, Italy, (IZSLER).

### 2.5 Fecal plate counting method

Fecal samples collected on d 0, d 28, d 56 from 12 calves (6 per treatment group) and analyzed using the plate counting method to evaluate the fecal bacterial count. Total bacterial, coliforms and *Lactobacillus* spp. were assessed using selective culture media, plate count agar (PCA), De Man, Rogosa and Sharpe agar (MRSA) and Violet Red Bile Lactose Agar (VRBLA), respectively (Liofilchem, Teramo, Italy). Each sample was processed using standard serial dilution technique (24, 25). Briefly, 1 g of feces was diluted in 9 mL of sterile physiological solution (NaCl 9%), and samples were diluted up to 10^−10^. For each selected dilution, 1 mL of diluted sample were plated in triplicate onto petri dishes at different dilutions in triplicate. The inclusion method was applied for total bacteria with an aerobic incubation, while *Lactobacillus* spp. and coliform an inclusion method plus e coverage layer was adopted to create a semi-anaerobic incubation. The total bacteria incubation period lasted for 3 days at 30 °C, the coliform incubation period lasted 24 h at 40 °C, and *Lactobacillus* spp. the incubation period lasted 3 days at 30 °C. Data are referred to log_10_ CFU/g considering the DM content of feces.

### 2.6 Ammonia and nitrous oxide quantification

Throughout the 56 days of the *in vivo* trial, the calves were housed on straw bedding, which was never fully replaced; only fresh straw was periodically added. At the end of the trial, animals were relocated to group housing, and manure (feces, urine, and straw) from each treatment group was collected, pooled, and transferred to six dynamic chambers (CTRL = 3; VITAN = 3) for ammonia and nitrous oxide quantification. Manure sampling within the pens was performed at five different depths and locations. The collected material was then pooled and transferred to the corresponding dynamic chamber for gas measurements. The manure was placed into 30 L dynamic chambers, which remained open throughout the trial and were closed only during measurements. The weight of each chamber was standardized (6.35 ± 0.96 kg), and manure temperature was recorded prior to each measurement (31.51 ± 5.42 °C). The variability in mass reflects the heterogeneous nature of the litter matrix (feces, urine, and straw) collected from the pens, which included variable amounts of straw bedding depending on daily replenishment during the 56-day trial. This variability is inherent to the practical conditions of commercial calf rearing and may have influenced gas emission measurements. All chambers were positioned in the same controlled location, and ambient temperature during the measurement period ranged from 11 to 28 °C. The gases quantification was observed on d 0, 1, 2, 5, 13, 34, 41, 49 using FTIR technique, Gasmet GT5000Terra. This system enabled simultaneous NH_3_ and N₂O analysis at a constant flow rate of 2 L/min. Each measurement cycle consisted of 5 min active measuring, in which 2 min in air to clean the probe after each measurement, and 3 min of sampling measurement in a hermetic closed container where the samples were inserted. And the raw data were converted from ppm to mg/g/h (Equation 1; (26)).


$$f(x)=\frac{\frac{\mathrm{\Delta c}}{\mathrm{\Delta t}}\times V\times p\times m}{R\times T\times g}\;$$ (1)

where: F = instantaneous gas flux (mg/g manure/h); Δc/Δt = rate of change in gas concentration during chamber closure (ppm s^−1^); V = chamber volume (m^3^); p = atmospheric pressure (Pa); m = molar mass of the gas (g mol^−1^); R = universal gas constant (8.314 J mol^−1^ K^−1^); T = air temperature inside the chamber (K); g = gram of matrix inside the chamber. Δc = change in gas concentration during the measurement period (ppm); Δt = duration of the measurement period (h).

The cumulative emissions (mg g^−1^) for each replicate (Cum_rep_) were estimated using the trapezoidal rule for numerical integration, as described in equation (Equation 2).


$${\mathrm{Cum}}_{\mathrm{rep}}=\sum_{\overset{`}{i}}\frac{F_{\overset{`}{i}\;\;}+F_{\overset{`}{i}+1\;\;}}{2}\times (\Delta t_{i,i+1}[h])$$ (2)

where: Cum_rep_ = cumulative emission per replicate, expressed as the product of flux and time (mg g^−1^); *F_i_*, *F*_*i* + 1_ = instantaneous gas fluxes measured at two consecutive sampling times *t*_i_, *t*_i + 1_ (mg g^−1^ h^−1^); *F_i_*, *F*_*i* + 1_**/**2 = average flux between two consecutive sampling times; Δ_ti,i + 1_ = time interval between two consecutive measurements, expressed in hours [h]; Σ = summation over all consecutive measurement intervals.

Samples were collected at the beginning and at the end of the trial to be analyzed for the content of total solids at 105 °C (TS; g), ash at 600 °C (%), total volatile solids (TVS; %), total nitrogen (TN; %), ammoniacal nitrogen (N-NH_3_; %), nitrates (%), and total carbon (TC; %), the begin (d 0), when the animals were moved and the manure was collected in the dynamic chamber, and at the end (d 49) of gas monitoring activity. TS were determined by oven drying at 105 °C until constant weight (AOAC 934.01). Ash content was measured by combustion at 550 °C in a muffle furnace (AOAC 942.05), and total volatile solids (TVS) were calculated by difference. TN was determined using the Kjeldahl method (AOAC 984.13). N–NH₃ and nitrates were analyzed according to Standard Methods (APHA, 2017). TC was determined by dry combustion using an elemental analyzer (ISO 10694).

### 2.7 Statistical analysis

Twelve animals for each group were included in the study, and the individual calf was considered as experimental unit. The sample size (*n* = 12 per group) was determined *a priori* based on a power analysis for the primary outcome (ADG) using a two-tailed t-test (*α* = 0.05, power = 0.90), assuming a minimum detectable difference of 0.10 kg/d and a standard deviation of 0.08 kg/d (16), which indicated that 7 animals per group were required. To account for potential attrition, 12 calves per group were enrolled.

Data analysis was conducted using SAS 9.4 and RStudio 4.3.3. Normality of residuals was assessed using Shapiro–Wilk test. Animal performance (MI, BW, HI, ADFI, ADG, RT) and plasma metabolic compound data were analyzed using generalized linear mixed models (PROC GLIMMIX, SAS 9.4). The model included treatment, time, and their interaction as fixed effects, with animal as a random intercept to account for repeated measures. Covariance structures were compared using AIC/BIC, with the variance components (VC) structure selected. Model diagnostics included examination of Pearson and studentized residuals, Q-Q plots for distributional assumptions, and assessment of influential observations (Cook’s D). LS-means with 95% CI were computed for all fixed effects; pairwise comparisons used Tukey’s adjustment. Relative frequencies of fecal scores were calculated, and differences among groups were evaluated using a cumulative link mixed model (CLMM, RStudio 4.3.3) designed for ordinal response variables. Treatment, time, and the treatment × time interaction were fitted as fixed effects in the model. A spatial power covariance structure was used to account for unequally spaced time points, and pairwise comparisons were adjusted using Tukey’s method (RStudio 4.3.3). Fecal bacterial counts were analyzed using the ARTool package in R, which applies aligned rank transformation (ART) to allow the use of nonparametric factorial mixed-effects models. N₂O and NH₃ emission data were summarized descriptively. Given that manure was pooled prior to allocation to the emission chambers, the chamber units were considered technical replicates; therefore, no inferential statistical analysis was performed for these variables.

The results were considered statistically significant at *p*-values <0.05 and data were reported as the mean ± SD or as LS-means ± SEM.

## 3 Results

### 3.1 Growth performance and starter intake

No significant differences were observed between VITAN and CTRL groups for MI, HI, BW, ADG, and RT (*p* > 0.05; Table 2). MI, HI, BW and ADG weekly increased in both VITAN and CTRL groups, in line with animals’ growth (*p* > 0.05). Tannin’s supplementation significantly reduced the overall ADFI of starter intake in VITAN group (CTRL = 0.28 ± 0.01 vs. VITAN = 0.22 ± 0.01, *p* < 0.01; Table 2). Although starter intake tended to be low in VITAN group throughout the trial, the differences in each period in the time x treatment interaction were not significant (*p* > 0.092). Notably, the reduction in starter intake in the VITAN group did not compromise animal performance, with no changes observed in BW or ADG.

**Table 2:** Calves performances in control group (CTRL) and chestnut tannin group (VITAN) during the d 56 trial. Results are expressed as least square means (LSM) and standard error of the means (SEM).

Variable	Least squares means		SEM	*p*-value		
	CTRL	VITAN		Treatment	Time	Interaction
MI (L)	6.44	6.37	0.03	0.132	<0.01	0.850
BW (kg)	51.76	50.85	0.45	0.160	<0.01	1.000
HI (kg)	0.03	0.03	0.00	0.401	<0.01	0.205
ADFI (kg/d)	0.28	0.22	0.01	<0.010	<0.01	0.092
ADG (kg/d)	0.58	0.56	0.02	0.510	<0.01	0.873
RT (°C)	38.84	38.78	0.03	0.167	<0.01	0.698

MI, milk intake; BW, body weight; HI, Hay intake; ADFI, average daily feed intake of starter intake, ADG, average daily gain; RT, rectal temperature. Treatment differences compared to the control group.

### 3.2 Fecal and clinical score evaluation

**Figure 2:**
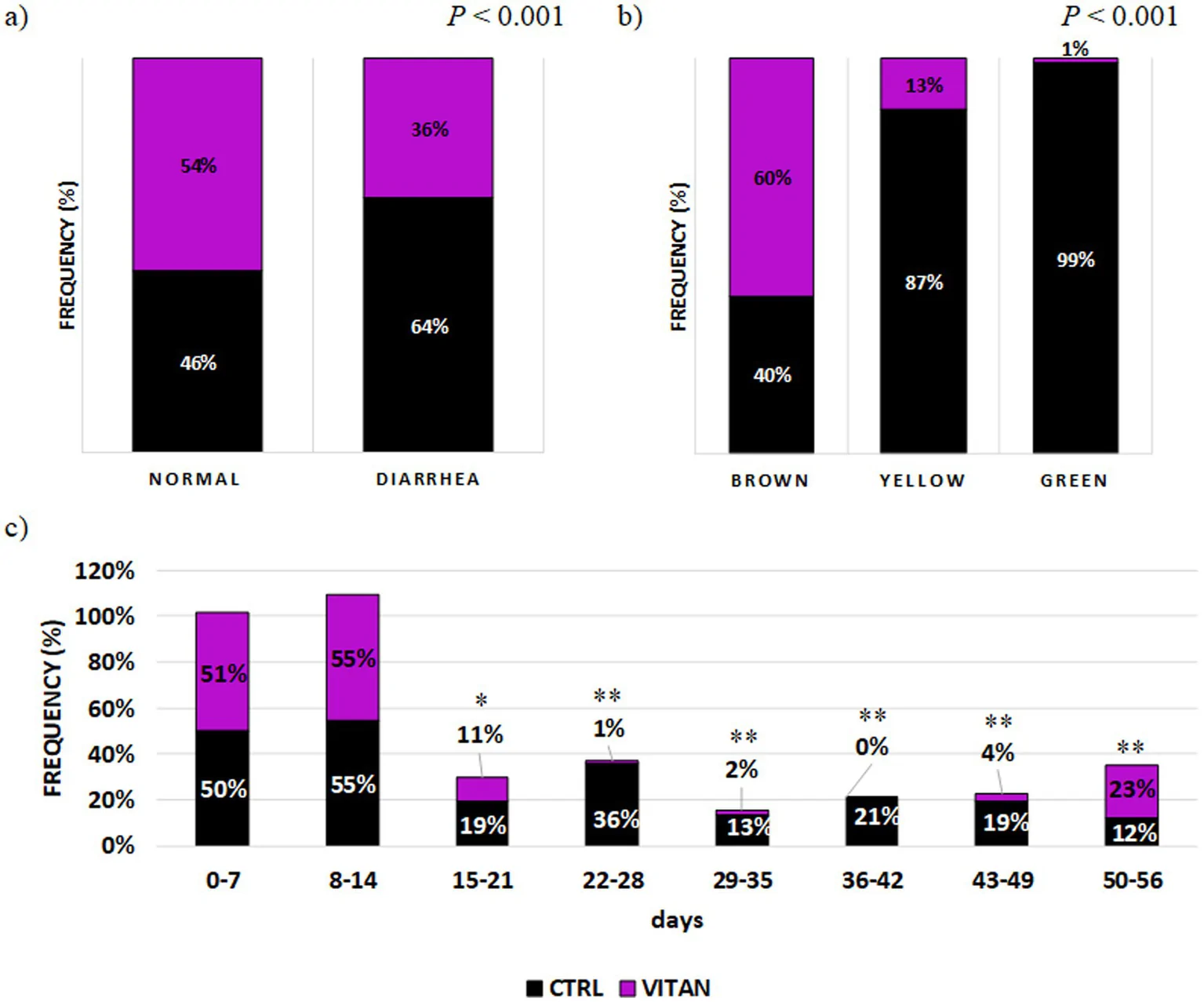
**(a)** Fecal consistency score frequency (%), the evaluation of consistency of feces. Normal = 0–1 score, diarrhea = 2–3 score. **(b)** Fecal color score frequency (%), brown, yellow, and green. **(c)** Frequency (%) of fecal scores 2–3 (diarrhea) within each treatment group (CTRL and VITAN) over time. Percentages are expressed relative to the total number of fecal observations within each group and time point, where total observations are defined as the sum of normal feces (scores 0–1) and diarrhea cases (scores 2–3). *p-value <0.01, **p-value <0.001.

The relative frequency (%) of daily fecal consistency and fecal color score showed that VITAN group presented lower frequency of abnormal fecal consistency (fecal scores 2–3; *p* < 0.01; Figure 2a). The frequency of fecal score consistency evidenced that fecal consistency score (FS) 0, 1, 2, 3 showed differences in frequency distribution between the two groups, the CTRL group showed a % of 1.46, 71.34, 24.12, 3.07, while VITAN group denoted a % of 43.86, 40.20, 12.87, 3.07, respectively. Fecal color scores result in high frequency of brown fecal color in the VITAN group, while the CTRL group showed a higher frequency of yellow feces (*p* < 0.01). The green color was observed only in the CTRL group (*p* < 0.01; Figure 2b). There was lower frequency of fecal score consistency on d 22–28, d 29–35, d 36–42, d 43–49, and 50–56 in VITAN group (FS 2–3; *p* < 0.01; Figure 2c).

Clinical signs represented by sunken eyes, low body condition, cough, and breathing presented no differences between the two groups throughout the trial (*p* > 0.05; Table 3). No significant differences were observed between groups for any clinical sign (sunken eyes, low body condition, cough, breathing; Table 3), indicating that the improved fecal consistency in the VITAN group was not associated with a reduction in clinically defined calf diarrhea, which typically includes dehydration, depression, and metabolic acidosis.

**Table 3:** Clinical sign frequency (%) between control group (CTRL) and chestnut tannin group (VITAN) during the d 56 trial.

Variable	Treatment	Scores					
		0	1	2	3	4	5
Breathing (%)	CTRL	97	3				
	VITAN	97	3				
Cough (%)	CTRL	100	0	0			
	VITAN	100	0	0			
Sunken eyes (%)	CTRL	91	8	1	0		
	VITAN	92	7	1	0		
Low body condition (%)	CTRL	86	11	3	0	0	0
	VITAN	84	14	1	1	0	0

Breathing: 0–1 score, cough: 0–2 score, sunken eyes: 0–3 score, low body condition: 0–5 score.

### 3.3 Plasma metabolites and feces plate counting method

At enrolment (day 0), all calves had total serum protein concentrations above the commonly accepted threshold for adequate passive transfer (>52 g/L). Mean values were 57.97 ± 2.21 g/L in the CTRL group and 62.92 ± 2.21 g/L in the VITAN group (Table 4). *γ*-globulin concentrations were 10.16 ± 1.61 g/L in the CTRL group and 14.12 ± 1.61 g/L in the VITAN group, both above the reference value (>10 g/L) indicative of successful passive immunity. Similarly, GGT values were 244.83 ± 79.38 IU/L in the CTRL group and 291.55 ± 79.38 IU/L in the VITAN group, consistent with adequate colostrum intake and absorption. These findings confirm that all calves entered the study with satisfactory passive immune status. Plasma metabolites did not differ between CTRL and VITAN groups along the trial, with no effects in treatment and time x treatment interaction (Table 4). All the parameters were within the reference intervals provided by IZSLER and reported in the literature for calves (27).

**Table 4:** Blood metabolic profile of preweaning calves in control group (CTRL) and chestnut tannin group (VITAN) on d 0 and d 56.

Variable	CTRL	VITAN	SEM	*p*-value
Total protein (g/L)				
d0	57.97	62.92	2.21	0.127
d56	61.93	60.78	2.21	0.954
Albumin (g/L)				
d0	29.49	28.96	0.69	0.864
d56	33.61	33.58	0.69	1.000
Globulin (g/L)				
d0	28.49	33.96	2.16	0.068
d56	28.32	27.20	2.16	0.953
γ globulin (g/L)				
d0	10.16	14.12	1.61	0.080
d56	8.63	7.02	1.61	0.747
Albumin/Globulin (ratio)				
d0	1.07	0.90	0.08	0.101
d56	1.20	1.24	0.08	0.948
Urea (mmol/L)				
d0	4.15	3.58	0.55	0.738
d56	2.32	2.22	0.55	0.998
Non-Esterified Fatty Acids (NEFA, mmol/L)				
d0	0.39	0.53	0.07	0.163
d56	0.37	0.41	0.07	0.947
Beta-Hydroxybutyrate (mmol/L)				
d0	0.06	0.05	0.02	0.821
d56	0.13	0.11	0.02	0.869
Glucose (mmol/L)				
d0	5.43	6.33	0.43	0.179
d56	5.65	5.23	0.43	0.758
Total cholesterol (mmol/L)				
d0	1.18	1.24	0.20	0.989
d56	3.17	2.94	0.20	0.671
High-Density Lipoprotein (HDL, mmol/L)				
d0	0.79	0.81	0.11	0.998
d56	1.97	1.85	0.11	0.675
Triglycerides (mg/dL)				
d0	0.18	0.25	0.04	0.196
d56	0.27	0.26	0.04	0.996
Aspartate Aminotransferase (AST-GOT, mmol/L)				
d0	51.33	56.58	7.18	0.884
d56	71.00	69.42	7.18	0.996
Gamma-Glutamyl Transferase (GGT, IU/L)				
d0	244.83	291.55	79.38	0.935
d56	26.50	40.33	79.38	0.998
Total Bilirubin (μmol/L)				
d0	13.79	18.14	2.60	0.349
d56	5.58	7.73	2.60	0.839
Creatine Kinase (CK, IU/L)				
d0	104.83	85.50	32.43	0.933
d56	174.33	203.33	32.43	0.808
Creatinine (μmol/L)				
d0	97.50	98.50	4.68	0.997
d56	66.25	68.58	4.68	0.959
Alkaline phosphatase (ALP, IU/L)				
d0	224.33	355.75	62.88	0.172
d56	314.92	374.67	62.88	0.778
Calcium (mmol/L)				
d0	3.13	3.12	0.08	0.998
d56	2.93	2.97	0.08	0.965
Phosphorus (mmol/L)				
d0	2.51	2.42	0.11	0.853
d56	3.12	3.03	0.11	0.885
Magnesium (mmol/L)				
d0	0.82	0.82	0.03	1.000
d56	0.86	0.83	0.03	0.711

Results are expressed as least square means (LSM) and standard error of the means (SEM).

**Figure 3:**
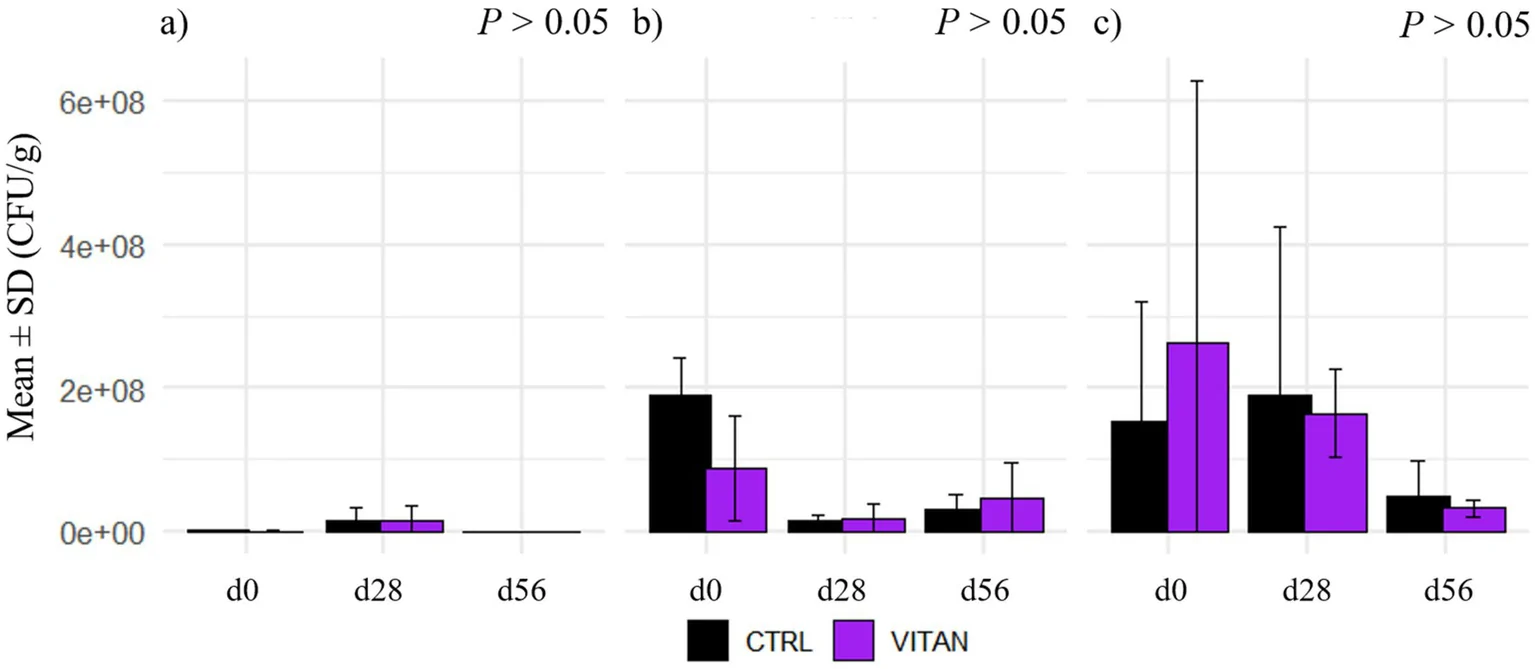
Fecal bacteria count (CFU/g) of **(a–c)** coliform bacteria, *Lactobacillus* spp., and total bacteria on day 0, day 28, and day 56. CRTL, Control group, VITAN: Treatment group. Data are expressed as mean ± standard deviation (SD).

Tannins did not affect fecal bacteria presence (Figure 3). No differences between groups for all the culture media has been observed, total bacteria and coliform presented similar quantities along all the trial, *Lactobacillus* spp. increased in both groups at the end of the trial. No significant treatment effects were observed for total bacteria (*p* = 0.215), *Lactobacillus* spp. (*p* = 0.279), or coliform bacteria (*p* = 0.470). Likewise, no significant treatment × time interactions were detected for total bacteria (*p* = 0.733), *Lactobacillus* spp. (*p* = 0.450), or coliform bacteria (*p* = 0.764). However, a significant effect of time was observed for *Lactobacillus* spp. (*p* = 0.002), whereas time effects for total bacteria (*p* = 0.088) and coliform bacteria (*p* = 0.255) were not significant.

### 3.4 NH₃ and N₂O emissions from calf manure

**Figure 4:**
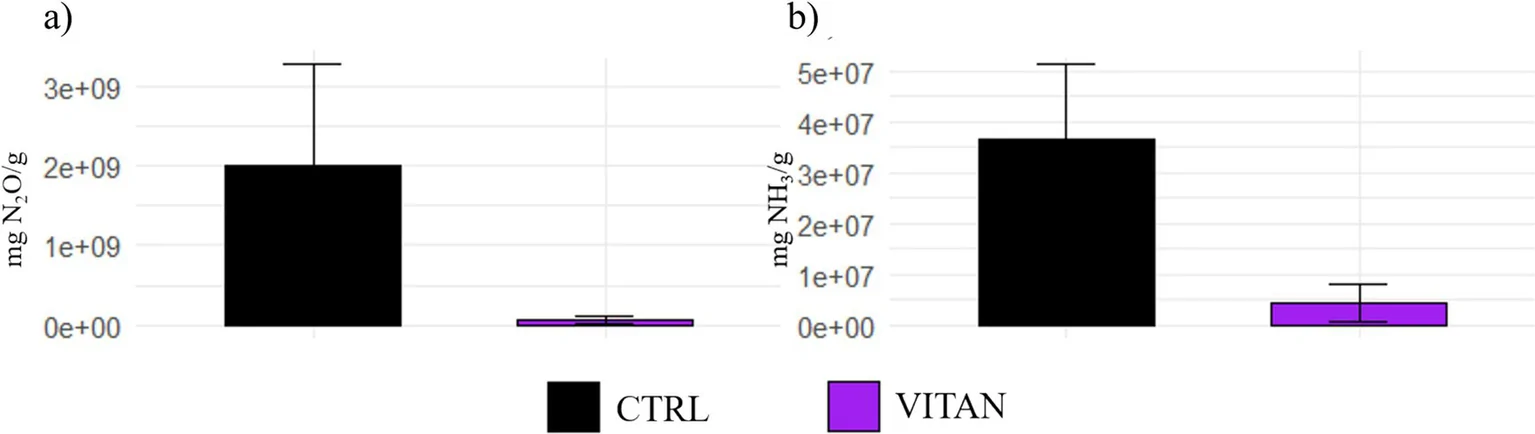
Cumulative emissions (mg/g) in VITAN and CTRL group of **(a)** N_2_O (mg/g) and **(b)** NH_3_ (mg/g). Data are expressed as mean ± standard deviation (SD).

**Figure 5:**
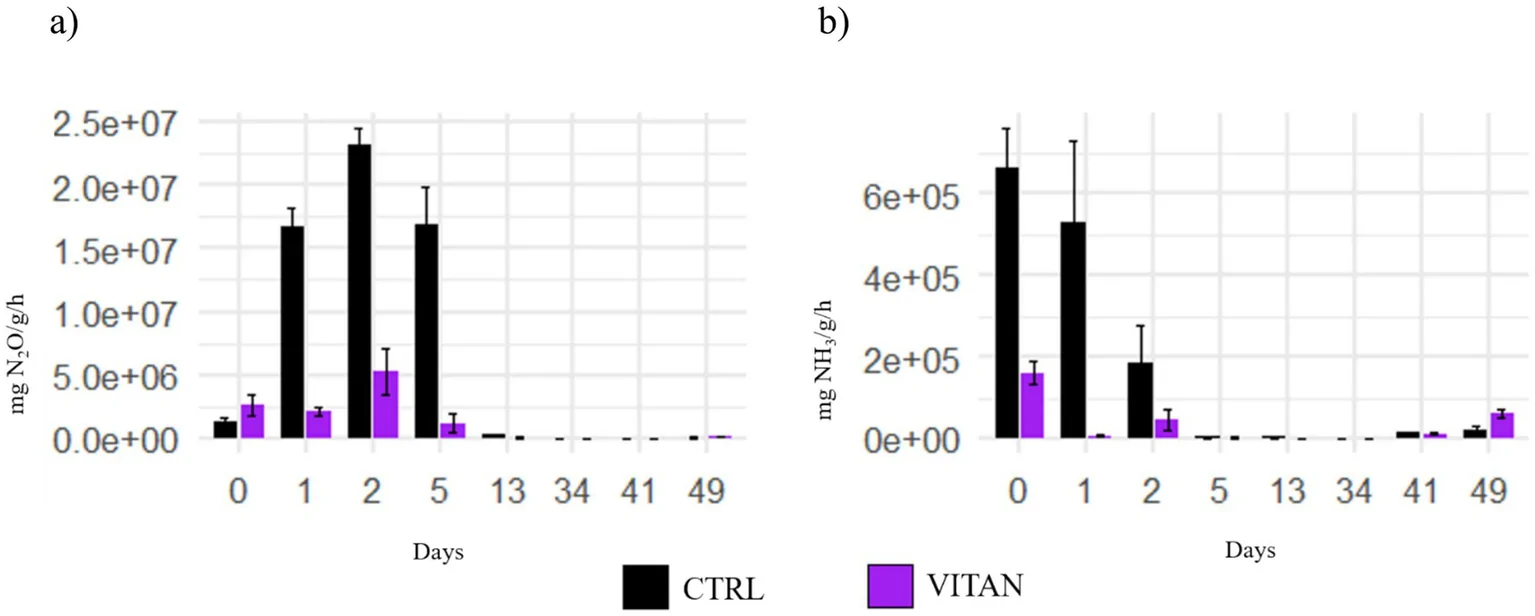
Mean and standard deviation of the instantaneous emission of **(a)** N_2_O and **(b)** NH_3_ over the days between CTRL and VITAN group (mg/g/h).

**Figure 6:**
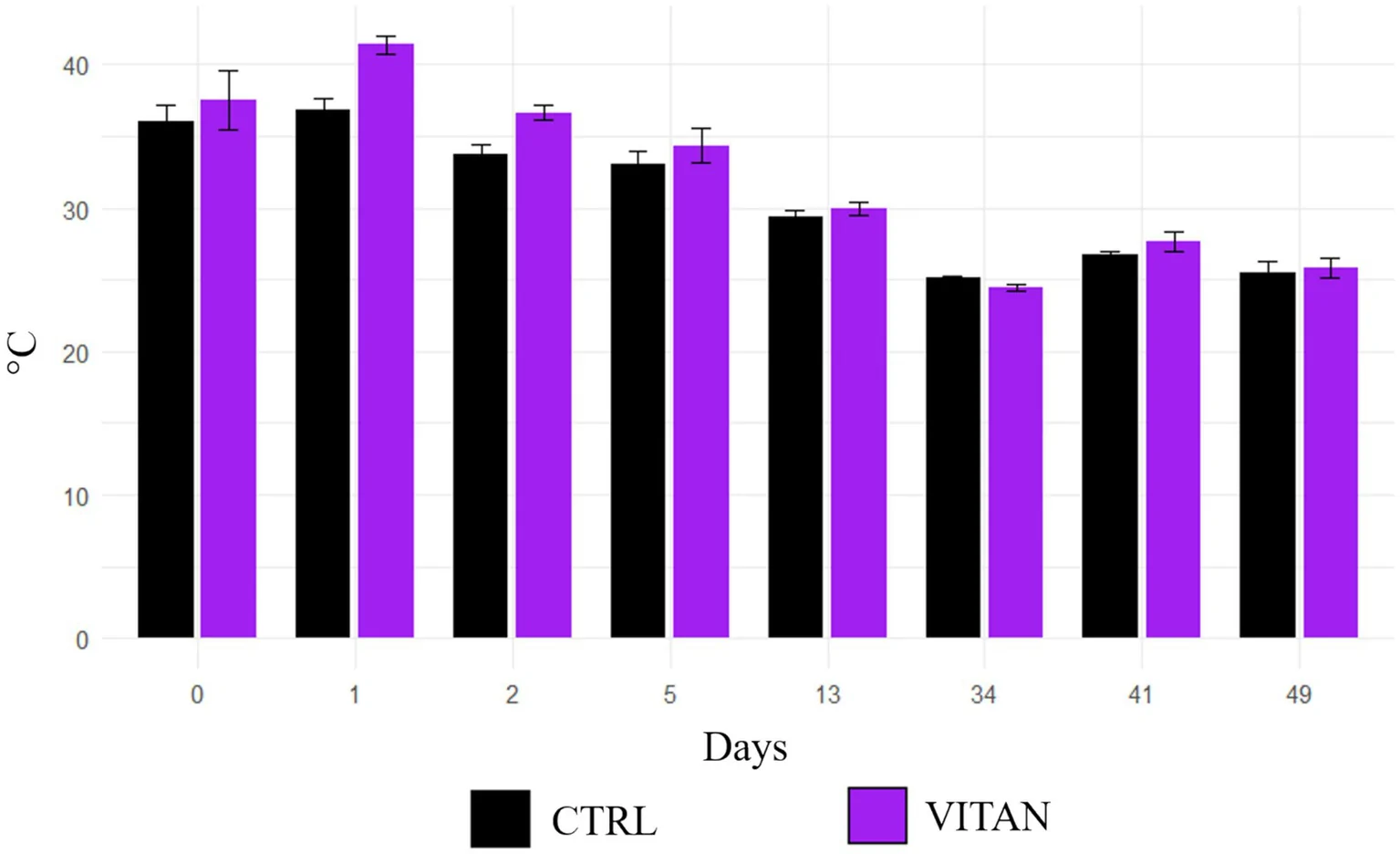
Mean and standard deviation of manure temperature (°C) of solid manure from preweaning calves’ manure in VITAN and CTRL group.

Cumulative emissions of N₂O and NH₃ were numerically lower in the VITAN group compared to the CTRL group (Figure 4). Treated group showed a decrease in N_2_O and NH_3_ emissions during the early storage phase (from d0 to d5) (Table 5; Figure 4a,b**)**. N_2_O emissions were lower in VITAN group on d 1, d 2 and d 5 (Figure 5a) and NH_3_ emissions were numerically lower in VITAN group on d 0 and d 1 (Figure 5b). Manure temperature in VITAN group was numerically higher on d 0, d 1, d 2 (Figure 6). The chemical analysis of manure evidenced an increase in the content of TS (%), ash (% of DM), TSV (% of DM), TN, nitrates, NH₄^+^-N and TC from d 0 (first measurement) to d 49 (last measurement; Table 6). Overall, TS showed an increase over time in both groups, with higher values observed in CTRL than in VITAN at the end of the experimental period. Ash content also seems to increase from d 0 to d 49, whereas TSV seems to have the opposite trend, decreasing in both treatments as the ash fraction increased. Total nitrogen numerically increased during the experimental period, with higher values observed in the VITAN group on d 49. In contrast, NH₄^+^-N concentrations markedly decreased from d 0 to d 49 in both groups, although values remained higher in VITAN than in CTRL at the final sampling. Nitrate concentrations seems to increase over time, particularly in the VITAN group. Total carbon decreased from d 0 to d 49 in both treatments, reaching similar values at the end of the trial. These observations are presented as descriptive statistics (gas emissions as mean ± SEM and chemical analysis of manure as mean ± SD) and should be considered preliminary due to the technical nature of the replicates.

**Table 5:** Cumulative emissions (mg/g/h) of nitrous oxide (N_2_O) and ammonia (NH_3_; mean ± standard deviation (SD), 95% CI).

Gas	TRT	Mean (Log_10_)	SEM (Log_10_)	CI low (Log_10_)	CI high (Log_10_)
N₂O	VITAN	8.30	6.56	8.26	8.33
	CTRL	9.52	8.43	9.33	9.65
NH₃	VITAN	6.88	6.44	0.00	7.29
	CTRL	7.56	6.62	7.25	7.73

Results are expressed as least square means (LSM) and standard error of the means (SEM).

**Table 6:** Chemical composition of manure between treatments (CTRL vs. VITAN) at d 0 and d 49, including total solids (TS), ash, total solids volatile (TSV), total nitrogen (TN), ammonium nitrogen (NH_4_^+^-N), nitrates, and total carbon (TC).

Parameter	Day	Mean ± SD	
		CTRL	VITAN
TS (%)	0	18.43 ± 0.21	21.59 ± 0.46
	49	34.81 ± 1.08	29.67 ± 0.17
Ash (%)	0	17.23 ± 0.39	13.97 ± 0.40
	49	25.28 ± 0.77	25.00 ± 0.36
TSV (%)	0	82.77 ± 0.39	86.03 ± 0.40
	49	74.72 ± 0.77	75.00 ± 0.36
TN (% DM)	0	2.66 ± 0.03	2.63 ± 0.09
	49	2.79 ± 0.04	3.49 ± 0.04
NH₄+-N (μg/g DM)	0	1371.70 ± 61.01	1512.30 ± 317.89
	49	558.40 ± 12.66	725.50 ± 17.33
Nitrates (μg N/g DM)	0	174.32 ± 41.77	144.64 ± 9.84
	49	180.28 ± 6.47	229.47 ± 10.31
TC (% DM)	0	48.01 ± 0.23	49.90 ± 0.23
	49	43.34 ± 0.45	43.50 ± 0.21

Data are expressed as mean ± standard deviation (SD).

## 4 Discussion

Tannin supplementation in calves did not affect body weight or average daily gain, indicating that overall growth performance was maintained. This finding is consistent with previous studies in both dairy cows and preweaning calves, where dietary inclusion of tannins did not impair growth (16, 28, 29). Interestingly, in a study on finishing lambs, average daily gain increased linearly with tannin supplementation, control animals showed lower ADG compared to lambs receiving 4 g/kg of tannins (30). RT was monitored for the detection of sickness in calves and to observe animal health; in healthy animals, it did not differ between the groups, consistent with previous findings (31). In line with the present study, Bonelli et al. (16) reported no interaction between tannins supplementation in milk intake (16), confirming that tannins may not affect the milk palatability and milk intake (32). Hay intake (HI) appears to reflect early dietary effects and is closely associated with future rumen development (33). Although hay intake remained low in the present study, early consumption of solid feed may still contribute to the adaptation of calves to future dietary transitions. The consumption of starter feed is considered a key driver of rumen development, as increased fermentation activity generates volatile fatty acids that act as signaling molecules regulating epithelial growth, papillae differentiation, and the transition toward a functional ruminant digestive system (34). The lower starter intake observed in the tannin-supplemented group, despite similar body weight between treatments, may suggest a potential improvement in feed efficiency and a possible reduction in nutrient excretion during the experimental period, which are relevant factors during animal growth (35). Calves, like monogastric animals, are not fully adapted to dietary tannin intake and generally exhibit a lower tolerance to the astringent effects associated with tannin–protein interactions. Increased astringency may negatively affect feed palatability and reduce feed intake (11). However, it is possible that tannin supplementation influenced digestive processes or postprandial satiety, indirectly affecting the motivation to consume solid feed. However, the mechanisms underlying this response remain unclear and warrant further investigation (36). The formation of tannin–protein complexes within the gastrointestinal tract may provide a protective effect, limiting oxidative degradation of nutrients such as proteins, carbohydrates, and lipids during digestion. Feed digestibility represents a key determinant of the physiological impact of tannins at the gastrointestinal level. In addition, the fate and absorption of condensed tannins remain more complex due to their greater structural variability and resistance to breakdown (11). The differences in feed intake between treatments are widely discussed in the literature, and the variability in results could be related to the chemical structure or molecular weight of different types of tannins, which may modulate feed intake (32). Overall, the effects of tannins on dry matter intake (DMI) appear to be both dose- and species-dependent. High inclusion levels of tannins in dairy cows (2.25% of DMI) have been associated with reduced intake, often in a linear manner as supplementation increased (28, 32). In contrast, lower inclusion rates did not affect DMI in piglets (1.25% of DMI; (29)), while moderate supplementation in lambs resulted in a linear increase in DMI (0.4 and 0.2% of DMI; (30)). These contrasting findings suggest that the impact of tannins on feed intake may vary according to animal species, physiological stage, dietary composition, and tannin type or dose.

All calves involved in the present study received a sufficient quantity and quality of colostrum, which was ensured by a Brix refractometer reading greater than 25%. This was further supported by objective indicators of passive immunity: total serum protein concentrations at enrolment were above the commonly accepted threshold for adequate passive transfer (>52 g/L), with a mean ± SD of 59.9 ± 4.2 g/L. *γ*-globulin concentrations were above 10 g/L in both groups, and GGT values were consistent with successful colostrum absorption (Gunes et al., 2022). These findings confirm that all calves entered the study with satisfactory passive immune status. Proper colostrum intake ensures longer-lasting passive immunity, which can reduce the incidence of acute diarrhea and respiratory diseases (37, 38). In the present study, tannin supplementation appeared to improve in fecal consistency. Calves in the VITAN group showed higher frequency of solid feces (FS = 0) and a lower frequency of moderate diarrhea (FS = 2; *p* < 0.01). Although no effect was observed on the most severe fecal consistency score (FS 3), the reduction in mild soft consistency may suggest a potential role of tannins in improving fecal consistency. These findings are consistent with previous studies suggesting that tannins exert effects primarily by modulating fecal consistency (16). This functional activity appears to be particularly relevant during the first weeks of life, when passive immunity declines and active immunity begin to increase (d 15–21; (38)). Dietary tannin inclusion has been associated with reductions in diarrhea incidence and duration in young ruminants, supporting their potential use as a functional ingredient to improve potential beneficial effects on gastrointestinal function during critical early-life periods (14, 16, 32, 39). However, not all studies have demonstrated clear reductions in clinically defined diarrhea following tannin or polyphenol supplementation in neonatal calves. Boccardo et al. (20) evaluated a polyphenol-rich hazelnut skin extract (5 g/day) in neonatal dairy heifers and found a reduction in the duration of watery feces, but no significant effects on overall clinical health issues associated with neonatal calf diarrhea, lung lesions, or weight gain. This finding is consistent with our results, as we observed improved fecal consistency without differences in clinical parameters between treatment groups. Several factors may explain these discrepancies. The composition and concentration of bioactive compounds vary substantially among products: the hazelnut skin extract used by Boccardo et al. (20) is a polyphenol-rich extract, whereas our study evaluated a purified chestnut tannin extract containing 224.8 mg/g of total hydrolysable tannins, with vescalagin (45.2 mg/g) and castalagin (39.7 mg/g) as the predominant compounds. In addition, the dosage and duration of supplementation differ across studies. Furthermore, the heterogeneity in case definitions of calf diarrhea (40) may contribute to variable outcomes. Therefore, the efficacy of tannin supplementation likely depends on the specific product composition, dosage, and study design. Our results in the fecal color evidenced high frequency of brown feces in the treatment group, potentially related to the presence of tannin, a brown additive. Fecal color could be influenced by feed intake during the first weeks of life. Initially, newborn calves consume only milk, resulting in more yellow feces, as calves begin to consume solid feed in addiction to milk, at 2–3 weeks of life, fecal color gradually changes (16). Green feces are considered abnormal in preweaning animals (41). In piglets, age and nutrition are the major factors that influence the color of feces and consequently the microbial population (42). Although it is not possible to determine whether the brown color was directly caused by tannins or indirectly by improved intestinal function, the fecal profile in treated calves aligns with healthier gastrointestinal status compared to controls. No significant differences in health-related parameters have been reported in calves-fed tannin-supplemented diets. Clinical scores of all animals remained at or near 0 throughout the experimental period, indicating overall good health status. Clinical sign scores were directly related with bovine respiratory disease and diarrhea phenomena (43). Sunken eyes score is a direct symptom of dehydration, and diarrhea could be the main responsible (17). Low body condition score is connected to low animal health, caused by multiple reasons, such as diarrhea (22), cough and breathing parameters were evaluated for the presence of respiratory disease that could influence the data observation, and often manifests as diarrhea (22). Consistent with the observations on clinical health parameters, no differences were found in plasma metabolites between the CTRL and VITAN groups, suggesting that tannin supplementation did not affect either physiological or health status in preweaning calves. Although tannins did not affect any blood metabolites in the present study, the available literature reports inconsistent results. Modulation of blood biochemical parameters has been observed in different species, particularly affecting protein and lipid metabolism indicators such as albumin, HDL, LDL, triglycerides, blood urea nitrogen, and other metabolic markers (14, 16, 29, 30, 44, 45). No significant differences were observed in fecal bacterial counts between the CTRL and VITAN groups, suggesting that tannin supplementation did not markedly affect either systemic physiological status or total bacteria composition in preweaning calves. Fecal bacterial quantification on d 0, d 28, d 56 provided an overview of the fecal bacteria presence during the preweaning period. These findings differ from those reported by Serri et al. (17), who observed a reduction in *Escherichia coli,* and an increase in *Lactobacillus* spp. following the supplementation of 4 g or 6 g of hydrolysable tannin, indicating a positive effect on gut health. Differences in tannin source or dosage may have influenced the fecal bacteria. The antibacterial effect of tannins has been studied in piglets and chickens, both in the small intestine and in colon, where they have shown to reduce gut damage caused by *Clostridium perfrigens* (14). In piglets infected with enterotoxigenic *Escherichia Coli* F4 (ETEC F4), tannins showed no effect on bacterial shedding (46). Fecal composition still plays a central role in determining manure composition and nutrient availability, which can influence gaseous emissions. For the manure emission experiment, manure (feces, urine, and straw) was pooled by treatment before chamber allocation. The three chambers per treatment therefore represent technical replicates. Additionally, the variability in litter mass (6.35 ± 0.96 kg), attributable to the straw component, may have influenced temperature, aeration, microbial activity, and gas diffusion processes. Consequently, the emission findings should be considered preliminary, and statistical inference is limited. In the present study, calves in the VITAN group exhibited lower NH₃ and N₂O emissions from the manure compared to the CTRL group under the experimental chamber conditions, although no marked differences were observed in fecal bacterial counts. This effect may be related to the ability of tannins to interact with dietary proteins, which could potentially reduce microbial protein degradation and ruminal ammonia production, thereby improve nitrogen utilization and decrease the release of volatile nitrogen compounds (47). The impact of tannins on NH₃ and N₂O emissions observed during early storage phase has been widely investigated in ruminants, although responses appear to depend on factors such as diet composition, dry matter intake, and animal age (48). Indeed, in literature tannins have been reported to influence NH_3_ and N₂O emissions from manure by modulating microbial fermentation and improving nitrogen utilization, thereby decreasing protein degradation and the release of volatile compounds (47, 49, 50), although their the effects of tannins on gas emissions are not always consistent. The reduction in NH₃ and N₂O emissions observed during early storage phase can be explained by the ability of tannins to bind dietary proteins, thereby reducing ruminal protein degradation and shifting nitrogen excretion from urine to feces. Because urinary nitrogen is the primary source of NH₃ volatilization and subsequent N₂O formation, whereas fecal nitrogen is largely organic and mineralized more slowly, this redistribution decreases the availability of reactive nitrogen for gaseous losses during manure storage (50). However, these findings should be interpreted with caution, as the present study represents a preliminary evaluation based on technical rather than biological replicates. Therefore, further studies including biological replicates are needed to confirm these results and better characterize the effects of tannin supplementation on gaseous emissions during manure storage. Some *in vitro* studies reported no differences in NH₃ and N₂O emissions from slurry of cattle fed tannin-containing diets, while others observed increased NH₃ emissions after prolonged incubation periods (51). These discrepancies may be related to differences in tannin source, exposure time, substrate composition, and microbial activity (52). Overall, the reductions in NH₃ and N₂O emissions observed during early storage phase in the VITAN group suggest that tannin supplementation may contribute to improved nitrogen utilization and positively influence environmental emissions of manure during the preweaning period. The chemical composition of manure changed during the trial, reflecting the progressive transformation of organic matter occurring in the manure. The increase in total solids and ash content, together with the decrease in volatile solids and total carbon, indicates an ongoing mineralization process in which organic compounds were progressively degraded by microbial activity (53). Total nitrogen slightly increased over time, particularly in the VITAN group, while NH₄^+^-N concentrations decreased in both treatments and nitrate concentrations increased. This pattern suggests the occurrence of nitrogen transformation processes within the manure, such as nitrification, in which ammoniacal nitrogen is progressively converted into more oxidized forms. The higher total nitrogen and nitrate concentrations measured in the VITAN group at the end of the experimental period suggest that a greater proportion of nitrogen was retained in the manure in more stable forms. This effect may be related to the ability of tannins to bind dietary proteins and form tannin–protein complexes that are less susceptible to microbial degradation in the digestive tract, thereby modifying nitrogen utilization and excretion patterns (47, 54). At the same time, the higher nitrate concentrations observed in the VITAN group may reflect changes in nitrogen transformation processes, which are involved in N₂O production pathways (55, 56). Further investigations are needed to elucidate the mechanisms of nitrogen transformation, particularly regarding nitrate dynamics and microbial pathways involved in nitrification and denitrification.

## 5 Conclusion

The supplementation of tannin in preweaning calves did not negatively affect growth performance, as body weight and average daily gain were comparable between the VITAN and CTRL groups throughout the trial. However, calves receiving tannins showed a lower solid feed intake without compromising productive performance, which may suggest a potential difference in feed intake patterns between treatments. Tannin supplementation may have contributed to a lower frequency of fecal consistency scores of 2–3, which are associated with lower fecal dry matter and poorer fecal consistency. This effect may be related to the ability of tannins to bind proteins and other intestinal components, thereby limiting water loss into the intestinal lumen and improving fecal consistency. However, no differences were observed in clinical health indicators, plasma metabolites and fecal bacteria abundance between treatments. From an environmental perspective, tannin supplementation was associated with reduced N₂O and NH₃ emissions from manure under the tested conditions, suggesting a potential mitigation effect on nitrogen-related gaseous losses. Changes in manure chemical composition over time were observed in both groups, reflecting ongoing manure transformation processes during storage.

This study has some limitations that should be acknowledged. Microbiological analyses were limited to culture-based enumeration and therefore did not capture the full complexity of the gut microbiota. Importantly, the improved fecal consistency observed in the VITAN group was not associated with changes in clinical or metabolic parameters, likely because the overall incidence of clinical signs was low, suggesting that the observed effects were limited to fecal consistency. Furthermore, the study was conducted on a single farm using a specific hydrolysable tannin product and dosage, which may limit the generalizability of the findings. Finally, the emissions assessment should be considered preliminary, as it was based solely on technical replicates. Future studies should include biological replicates, comprehensive gut microbiota analyses, and validation under commercial farming conditions to confirm these findings.

Concluding, dietary tannin supplementation in milk replacer-fed calves may improve fecal health and reduce nitrogen-related emissions from manure without impairing animal performance, suggesting its potential as a nutritional strategy to enhance both animal health and environmental sustainability in calf rearing systems.

## Data Availability

The original contributions presented in the study are included in the article/supplementary material, further inquiries can be directed to the corresponding author.
